# Colon Mucinous Adenocarcinoma in Childhood: A Case Report with Emphasis on Image Findings

**DOI:** 10.1155/2010/327634

**Published:** 2010-03-25

**Authors:** Antonio Muccillo, Edson Marchiori, Cláudia Renata Penna, Regina Rodrigues Guimarães, Gláucia Zanetti, Guilherme Abdalla, Nina Ventura, Carolina Lamas Constantino, Mariana Leite Pereira, Viviane Brandão, Pedro Martins, Rodrigo Canellas, Romulo Varella de Oliveira

**Affiliations:** Department of Radiology, Federal University of Rio de Janeiro, CEP 21941.913. Ilha do Fundão, Rio de Janeiro, Brazil

## Abstract

Colorectal cancer is extremely rare in children. We report a case of a 12-year-old boy who presented with a five-month history of weight loss and anorexia, associated with vomiting episodes, dizziness, fatigue, and dyspnea. On physical examination, a palpable abdominal mass was noticed on the right hypochondrium and flank. An imaging study was performed, which showed a solid mass on the right colon. The patient underwent incisional surgical biopsy, and subsequent histopathologic analysis revealed a colon mucinous adenocarcinoma.

## 1. Introduction

Pediatric colon adenocarcinoma represents less than 1% of all neoplasms in the first two decades of life [1, 2]. Histologically, the aggressive mucinous carcinoma subtype accounts for most cases of this cancer [1, 3–5]. Although colorectal carcinoma has a good prognosis in adults when diagnosed early, in children, the rarity of the tumor and its high potential for dissemination usually lead to late diagnosis and poor prognosis [1–5]. In this report, we describe a case of colon adenocarcinoma in a 12-year-old boy, with emphasis on the imaging findings.

## 2. Case Report

A 12-year-old boy presented with a five-month history of significant weight loss (5 kg in one month) and anorexia, associated with vomiting, dizziness, weakness, and dyspnea. On physical examination, a palpable abdominal mass was noticed on the right hypochondrium and flank. The patient was also slightly pale, and a systolic murmur was observed by cardiac auscultation. Blood tests showed microcytic and hypochromic anemia and eosinophilia as the only abnormalities. The patient was negative for both acid-alcohol resistant bacillus and human immunodeficiency virus.

**Figure 1:**
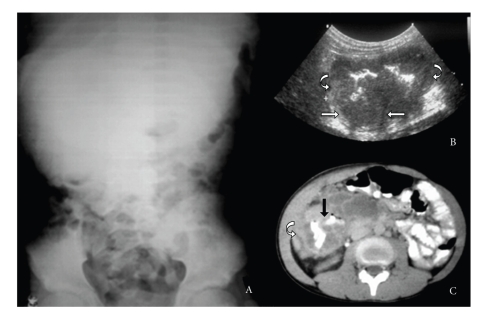
Abdominal X-ray (A) showing a soft-tissue mass with no calcification, on the right flank and mesogastrium, with inferior deviation of the bowel. Abdominal US (B) demonstrates a hypoechoic stenosing mass (curved arrow) measuring about 7.7 centimeters at greatest diameter, in the right colon. Enlarged mesenteric lymph nodes were also identified (arrows). CT of the abdomen (C) confirmed the presence of an infiltrating tumor, slightly enhanced by the contrast media (curved arrow), extending from the cecum to the hepatic flexure on the right colon wall, causing bowel lumen reduction and irregularity (black arrow). The adjacent meso and abdominal wall were also involved.

An abdominal X-ray showed a soft-tissue mass with no calcification, on the right flank and mesogastrium, with inferior deviation of the bowel (Figure 1A). Based on these findings, an ultrasonography (US) exam was performed, showing a hypoechoic stenosing mass of about 7.7 centimeters at the greatest diameter, in the right colon. Enlarged mesenteric lymph nodes were also identified (Figure 1B). 

Based on the imaging findings, computed tomography (CT) was performed on the abdomen. The scan confirmed the presence of an infiltrating tumor, with slight enhancement from the contrast media, located on the right colon wall, extending from the cecum to the hepatic flexure. The adjacent meso and abdominal wall were also involved. Mesenteric hypodense masses that might correspond to neoplastic implants were observed (Figure 1C). Chest and brain CT scans were normal. A colonoscopy demonstrated a stenosing and infiltrative blastomatous lesion in the right colon, blocking the equipment passage. Based on these findings, an incisional surgical biopsy was performed, and pathological analysis showed epithelial cells associated with numerous signet-ring cells inside mucinous lakes, consistent with mucinous adenocarcinoma. 

**Figure 2:**
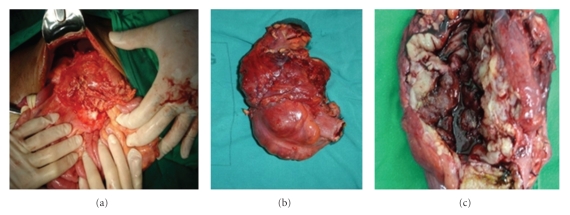
Surgery images. Tumor on the ascendant colon wall, extending from the cecum to the hepatic flexure.

The patient underwent a right hemicolectomy, with an end-to-end anastomosis between the ileum and the transverse colon (Figure 2). During the procedure, an unresectable macroscopic residual tumor was left near the emergence of the superior mesenteric artery, preventing a curative resection. Two enlarged mesenteric lymph nodes with macroscopic involvement were also found. No peritoneal effusion or macroscopic liver metastases were found. The patient underwent adjuvant chemotherapy (5-fluorouracil, Leucovorin, and Oxiplatin) and radiotherapy, but died 16 months after surgery.

## 3. Discussion

Colon adenocarcinoma is a rare tumor in pediatric patients, with an incidence of one in one million [1, 2]. This causes a low index of suspicion in physicians. Pediatric colon adenocarcinoma is associated with poorly differentiated histological type, and neoplasms are usually at an advanced stage at presentation, with liver and lymph node metastasis and in some cases, peritoneal carcinomatosis [1–5]. In this case, residual disease remained after surgery, with macroscopic lymph node involvement. 

Histologically, mucinous adenocarcinoma is the most common histotype in pediatric colon cancer [1, 3–5]. The literature is not clear about the tumor distribution in the colon; however, the disease does not appear to have a predisposition for any particular portion of the colon [1–4]. In this case, the lesion was in the right colon. The clinical features are similar to adults, with abdominal pain, altered bowel habits, weight loss, rectal bleeding, abdominal palpable mass, and anemia [1–6]. Our patient also presented with dizziness, weakness, dyspnea and anorexia, but no bleeding. Although mucinous adenocarcinoma can be a complication of familial polyposis of the colon and ulcerative colitis, most cases occur in children with no predisposing factors [5, 6]. According to published reports, the lag time between the onset of symptoms and diagnosis is usually 3 to 6 months and in general, patients present with several symptoms [1, 3].

Barium enema studies suggest that colon carcinomas in adults are generally small polypoid masses (early carcinoma), irregular annular lesions with abrupt margins, or large intraluminal masses [7–9]. By US abdomen examination, bowel wall thickening from colorectal carcinoma may result in a target or pseudokidney configuration, in which the central echogenic mucosa is surrounded by an abnormally thick hypoechoic rim [7]. Carcinoma should be suspected if a CT scan shows a discrete mass or focal thickening of the colonic wall. In advanced stages, a large soft-tissue mass containing calcification might be found, causing bowel obstruction. Evident wall thickening, enlarged lymph nodes, surrounding organs or mesenteric fat invasion, hydronephrosis, liver metastasis (seen as focal calcifications), and peritoneal effusion may also be noted. Other complications including perforation, abscess, and fistulas may also be observed [7, 9–11]. 

In this case, disease was at advanced stage at diagnosis, with CT and US exams showing a large mass in the right colon wall, causing significant reduction and irregularity of the lumen, and infiltrating the mesenterium and adjacent abdominal wall, consistent with the literature. Enlarged lymph nodes were also identified.

## 4. Conclusion

Prognosis in colorectal cancer is usually determined by the degree of intestinal wall invasion, lymph node involvement, and hematogenous metastases. For this reason, early detection increases the chance of cure. Therefore, the rarity, low level of suspicion, and aggressive histological type normally seen in children can lead, in most cases, to late diagnosis and poor prognosis.

In our case, imaging revealed extensive metastatic disease, disseminated throughout the peritoneal cavity, reflecting the delay in diagnosis that is described in the literature in similar cases. These findings were confirmed during surgery, which determined the impossibility of curative resection, and led to a poor prognosis, despite adjuvant therapy. 

## References

[1] Kravarusic D, Feigin E, Dlugy E (2007). Colorectal carcinoma in childhood: a retrospective multicenter study. *Journal of Pediatric Gastroenterology and Nutrition*.

[2] Chantada GL, Perelli VB, Lombardi MG (2005). Colorectal carcinoma in children, adolescents, and young adults. *Journal of Pediatric Hematology/Oncology*.

[3] Hill DA, Furman WL, Billups CA (2007). Colorectal carcinoma in childhood and adolescence: a clinicopathologic review. *Journal of Clinical Oncology*.

[4] Sebbag G, Lantsberg L, Arish A, Levi I, Hoda J (1997). Colon carcinoma in the adolescent. *Pediatric Surgery International*.

[5] Angelini C, Crippa S, Uggeri F, Bonardi C, Sartori P, Uggeri F (2005). Colorectal cancer with neuroendocrine differentiation in a child. *Pediatric Surgery International*.

[6] Karnak I, Ciftci AO, Senocak ME, Buyukpamukçu N (1999). Colorectal carcinoma in children. *Journal of Pediatric Surgery*.

[7] Kelvin FM, Maglinte DDT (1987). Colorectal carcinoma: a radiologic and clinical review. *Radiology*.

[8] Levine MS, Rubesin SE, Laufer I, Herlinger H (2000). Diagnosis of colorectal neoplasms at double-contrast barium enema examination. *Radiology*.

[9] Buetow PC, Buck JL, Carr NJ, Pantongrag-Brown L (1995). Colorectal adenocarcinoma: radiologic-pathologic correlation. *Radiographics*.

[10] Freeny PC, Marks WM, Ryan JA, Bolen JW (1986). Colorectal carcinoma evaluation with CT: preoperative staging and detection of postoperative recurrence. *Radiology*.

[11] Moss AA (1989). Imaging of colorectal carcinoma. *Radiology*.

